# Neuromodulation approaches and applications in the management of post-stroke pain: a comprehensive review

**DOI:** 10.3389/fneur.2026.1789715

**Published:** 2026-04-08

**Authors:** Stewart S. Cox, Marion Wood, Falon Sutton, Katherine Tucker, Nicole Cash, Ruxue Gong, Nathan C. Rowland, Steven A. Kautz, Mark S. George, Jeffrey J. Borckardt, Xiaolong Peng

**Affiliations:** 1 Department of Psychiatry and Behavioral Sciences, College of Medicine, Medical University of South Carolina, Charleston, SC, United States; 2 Department of Neurosurgery, College of Medicine, Medical University of South Carolina, Charleston, SC, United States; 3 MUSC Institute for Neuroscience and Discovery, Medical University of South Carolina, Charleston, SC, United States; 4 Department of Health Sciences and Research, College of Health Professions, Medical University of South Carolina, Charleston, SC, United States; 5 Ralph H. Johnson VA Medical Center, Charleston, SC, United States; 6 Division of Physical Therapy, Department of Rehabilitation Sciences, College of Health Professions, Medical University of South Carolina, Charleston, SC, United States

**Keywords:** central post-stroke pain, deep-brain stimulation, neuromodulation, post-stroke pain, transcranial magnetic stimulation

## Abstract

**Introduction:**

Post-stroke pain (PSP) remains a common and profoundly debilitating consequence of stroke, both in terms of a delay in recovery and in substantially reducing quality of life. Both invasive and non-invasive brain stimulation techniques are increasingly being explored as possible treatment modalities for various forms of PSP. This literature review examines the current body of evidence for all forms of neurostimulation for PSP.

**Methods:**

In this paper, we provide a review of the most recent literature exploring neuromodulation for PSP, covering several key domains: an examination of various PSP subtypes and the underlying mechanisms; a consolidation to date of the literature examining both invasive and non-invasive neuromodulation techniques for forms of PSP, and a discussion of future directions for the field.

**Results:**

The impact of neuromodulation techniques on PSP populations, focusing primarily on spasticity and central post-stroke pain (CPSP) is discussed.

**Conclusion:**

To varying degrees, numerous invasive and non-invasive modalities are beginning to be explored for individuals suffering from PSP. While preliminary, there is promising evidence to suggest that neuromodulatory techniques may reduce or ameliorate PSP. Further evidence and large clinical trials are needed to compare these treatments to the standard of care, as well as each other, to optimize outcomes for patients. In a rapidly evolving field, this review helps to provide the current state of neuromodulation in research on PSP.

## Introduction

Stroke is one of the most common and disabling neurological disorders. It affects nearly 800,000 individuals each year in the United States alone, with the number is expected to continue rising in the coming decades (1, 2). It is also responsible for a substantial reduction in quality of life and disability-adjusted life years as stroke survivors are burdened with multiple chronic issues such as motor and sensory deficits, cognitive impairment, and pain (3–6). Data suggest that nearly 70% of stroke survivors experience some form of post-stroke pain (PSP), a broad term that encompasses all pain syndromes that occur after stroke, which can include spasticity, complex regional pain syndrome (CRPS), and central post-stroke pain (CPSP). PSP can also negatively impact rehabilitation and delay recovery while simultaneously making recovery more expensive (7).

The current standard of care for PSP includes a multimodal approach of physical/occupational therapies and medications. While physiotherapy can be beneficial for improving of mobility and reducing pain, it can face limitations from patients’ chronic motor deficits or cognitive impairments following stroke, and there is currently limited knowledge on its impact on changes in pain intensity (5, 8). Medications may be helpful in some cases of PSP, but many of the medications, including antidepressants like amitriptyline, anti-inflammatories, and anti-epileptic drugs such as gabapentin and pregabalin, have demonstrated limited or mixed efficacy (5, 6, 9, 10). Further, many of these patients who are treatment-resistant to initial therapy require opioids for pain management, which can lead to dependency (11, 12). Therefore, there is growing interest and need for interventions that can attenuate chronic pain following stroke and improve patients’ quality of life. One such emerging intervention is neuromodulation via multiple brain stimulation techniques.

Neuromodulation techniques, broadly, are those that utilize methods to modify neural activity in a targeted manner to address various neurological conditions. An increasing number of studies indicate that invasive techniques, such as deep brain stimulation (DBS), motor cortex stimulation (MCS), spinal cord stimulator (SCS), and vagus nerve stimulation (VNS), may offer therapeutic benefit in treatment-refractory cases (13–15). While these techniques have shown analgesic properties in a range of neuropsychiatric conditions, including stroke, they are often not feasible options for individuals due to their expense, as well as being invasive surgical procedures (16–18). As such, non-invasive, affordable options are in great demand and are beginning to gain more traction. Transcranial magnetic stimulation (TMS), transcranial direct current stimulation (tDCS), transcutaneous auricular vagus nerve stimulation (taVNS), and low-intensity focused ultrasound (LIFU) are all generally safe, well-tolerated, affordable non-invasive modalities that, while additional research is still needed, can be beneficial for reducing chronic pain and improving quality of life in stroke patients (18). In the following sections, we will discuss the various types of PSP, the current literature on both invasive and non-invasive neuromodulation techniques for its treatment, proposed mechanisms of action, and future directions for the field.

For this narrative review, we conducted a targeted search of the literature to identify studies evaluating neuromodulation for the treatment of post-stroke pain. The primary search was performed in PubMed, covering the period from 1990 to the present. Search terms were constructed around key concepts related to post-stroke pain (e.g., “post-stroke pain,” “central post-stroke pain,” and “spasticity”) and combined with modality-specific terminology for each intervention (e.g., ((“deep brain stimulation” [Title/Abstract] OR “DBS” [Title/Abstract]) AND (“post stroke pain” [Title/Abstract]) OR (“central post-stroke pain” [Title/Abstract]) OR (“spasticity” [Title/Abstract])). In total, 683 publications were initially identified (DBS: 104; MCS: 62; SCS: 140; VNS: 9; TMS: 287; tDCS: 72; taVNS: 3; LIFU: 6). Advanced filters were used to exclude review articles, focusing only on clinical trials, randomized controlled trials, and meta-analyses. With this filter applied, 131 publications were identified. Eligible publications included studies in which at least one of the above neuromodulation modalities was applied to a form of post-stroke pain. Studies in which neuromodulation was delivered primarily in the context of motor rehabilitation or functional recovery (i.e., not targeting pain outcomes) were excluded. Relevant primary research articles were identified through a supplementary manual review of references from selected articles and prior reviews. Publications were screened for redundancy and relevance to the review topic, with emphasis on studies reporting therapeutic outcomes, mechanistic insights, or emerging applications of neuromodulation in post-stroke pain. In total, 31 articles were included. Of these articles, study rigor was assessed using an unweighted composite score based on sham control, sample size, and study design. Sham-controlled studies received 2 points, studies with a comparator but no sham received 1 point, and uncontrolled studies received 0 points; sample size was scored as 2 points for n ≥ 50, 1 point for n = 40–49, and 0 points for n < 20. Study design was scored as 2 points for randomized controlled trials, 1 point for prospective non-randomized or cohort studies, and 0 points for retrospective studies, case series, or case reports. Total scores were categorized as high rigor (5–6), moderate rigor (3–4), or low rigor (0–2) (see Tables 1, 2).

**Table 1:** Literature investigates invasive brain stimulation techniques for post-stroke pain.

Author, Year	Stim	Pain	Target	Parameters	Outcome measures	Findings	AEs	Rigor Score
Nowacki et al., 2025 (47)	DBS *n* = 39	CPSP	Posterior limb of the IC Sensorimotor thalamus Medial or intralaminar thalamus	Sham controlled: No Frequency: 1000 Hz Amplitude: NR Pulse Width: 90 μs Duration: 12 mo	NRS Volumes of tissue activated	DBS for medication-refractory chronic CPSP clinically effective in 36% of patients	No	1
Niu et al., 2024 (46)	DBS *n* = 1	CPSP	Thalamus	Sham controlled: No Frequency: 15, 75, 130 Hz Amplitude: 2–4.75 V Pulse Width: 50, 60, 90 μs Duration: 7 mo	Thalamic local field potential activity in relation to pain Multi-day local field potential recordings from left ventral posterolateral nucleus	Power spectral density varied with pain levels in delta, theta, and gamma bands DBS modulated pain-related thalamic oscillations; power spectral density changes may serve as quantitative indicators of pain relief	No	0
Boccard et al., 2013 (53)	DBS *n* = 85	NP	PVG Ventral posterior nuclei of thalamus	Sham controlled: No Frequency: 5–50 Hz Amplitude: 0.5–0.5 V Pulse Width: 200–450 μs Duration: Mean 19.6 mo	VAS Quality-of-life survey McGill pain questionnaire EuroQol-5D questionnaires	>30% improvement in VAS & McGill Pain Questionnaire >30% improvement in Short-Form 36 Questionnaire and EuroQol-5D Questionnaire Some improvement maintained at 1-year follow-up	No	3
Mallory et al., 2012 (65)	DBS *n* = 1	CPSP	Nucleus accumbens PVG	Sham controlled: No Frequency: 130 Hz Amplitude: 1–4 V Pulse width: 90–450 μs Duration: 11 mo	VAS	VAS pain rating improved up to 11-month follow-up Pain recurred if electrodes turned off at either site	No	0
Lempka et al., 2017 (68)	DBS *n* = 9	PSP	VS ALIC	Sham controlled: Yes Frequency: 130 Hz Amplitude: 1–4 V Pulse width: 90, 210 μs Duration: 24 mo	Beck Depression Inventory Beck Anxiety inventory Columbia-Suicide Severity Rating Scale Montgomery-Asberg Depression Rating Scale Positive and Negative Affect Schedule Pain Disability Index VAS Short-Form McGill Pain Questionnaire	No significant difference in pain disability or VAS between DBS ON and OFF Greater improvement in depression symptoms with DBS ON Improvement in affective pain experience with DBS ON No change in McGill score	Yes	4
Gopalakrishnan et al., 2018 (69)	DBS *n* = 10	CPSP	VS	Sham controlled: Yes Frequency: 100, 130 Hz Amplitude: 2–6 V Pulse width: 90, 210 μs Duration: 24 mo	MEG event-related fields for anticipation of pain	Decrease early N1- restored salience & discrimination Decrease posterior P2 - reduced anticipatory anxiety Increase anterior N1 - improved cognitive/emotional regulation (in responders)	No	4
Jones et al., 2021 (70)	DBS *n* = 5	PSP	VS ALIC	Sham controlled: Yes Frequency: 100, 130 Hz Amplitude: 1–6 V Pulse width: 60, 90, 120 μs Duration: 24 mo	fMRI BOLD Heat stimulus	Reduced activation in cortical areas Decreased activation in hippocampi during painful stimulation to affected side	No	4
Franzini, A. et al., 2020 (71)	DBS *n* = 4	CPSP	IC	Sham controlled: No Frequency: 100 Hz Amplitude: 1.5–2 V Pulse width: 60 μs Duration: Mean 5.88 yrs	VAS (long-term intensity & satisfaction with stimulation)	Pre-op VAS: 9 (range 8–10) Short-term follow-up (1 week): VAS 3 (range 0–6) Long-term (mean 5.88 y): VAS 5.5 (range 3–8)	No	0
Guo et al., 2022 (75)	MCS *n* = 21	CPSP	M1	Sham controlled: No Frequency: 30–50 Hz Amplitude: 3.5–7 V Pulse width: 210–300 μs Duration: Mean 65.43 mo	VAS Neuropathic Pain Symptom Inventory Pittsburgh Sleep Quality Index	Thalamic stroke group: Decrease in VAS pain scores Reduction in neuropathic pain Improved sleep quality Extra-thalamic stroke group: Modest VAS pain reduction	No	1
Zhang et al., 2017 (76)	MCS *n* = 16	CPSP	M1	Sham controlled: No Frequency: 10 Hz Amplitude: 0.8–2.4 V Pulse Width: 450–500 μs Duration: Mean 28 mo	VAS	VAS significantly decreased from baseline at 1 month follow-up, which was maintained at 28 months	Yes	0
Zhang et al., 2018 (77)	MCS *n* = 16	CPSP	M1	Sham controlled: No Frequency: 30–50 Hz Amplitude: 3.5–7 V Pulse width: 210–300 μs Duration: Mean 28.2 mo	VAS Neuropathic Pain Symptom Inventory	Decrease in VAS pain score Decrease in total Neuropathic Pain Symptom Inventory score Relief of burning pain associated with positive outcomes	No	0
Lefaucher et al., 2011 (83)	MCS *n* = 6	CPSP	M1	Sham controlled: Yes Frequency: 40 Hz Amplitude: 2 V Pulse width: 60 μs Duration: 12 mo	VAS Verbal Rating Scale Brief Pain Inventory McGill Pain Questionnaire Sickness Impact Profile Medication Quantification Scale	Randomized phase: All clinical scores decreased in MCS ON vs. OFF Open follow-up: Significant improvement in all scores Medication Quantification Scale: greater effect on affective component of pain	Yes	4
Busch et al., 2024 (86)	SCS *n* = 2	CPSP	Spinothalamic tract	Sham controlled: No Frequency: 50 Hz Pulse width: 1000 μs Duration: NR	NRS	Both patients had pain resolution following BurstDR SCS	Yes	0
Zhang et al., 2024 (88)	SCS n = 1	CPSP	Cervical	Sham controlled: N Frequency: 40 Hz Pulse width: 450–500 μs Duration: 5 mo	NRS	Approximately 75% pain reduction following stimulation compared to baseline	No	0
Hosomi et al. 2022 (89)	SCS *n* = 166	CPSP	Cervical Lower thoracic Midline cervical Upper cervical	Sham controlled: No Frequency: 25–50 Hz Pulse width: 210 μs Duration: Median 24 mo	NRS Patient Global Impression of Change scale	Mean pain score decreased 40% immediately after stimulation and maintained until follow-up (24–63 months) Around 60% of patients had ≥30% pain reduction at both time points	Yes	2
Tanei et al., 2023 (90)	SCS *n* = 1	CPSP	Cervical spine 3–5 Thoracic spine 8–9	Sham controlled: No Frequency: 90 Hz Pulse width: 210 +/− 50 μs Duration: Mean 67.3 mo	NRS Pain Catastrophizing Scale	NRS pain decreased Pain Catastrophizing Scale decreased	No	0

AEs, adverse events; ALIC, anterior limb of internal capsule; CPSP, central post-stroke pain; DBS, deep brain stimulation; IC, internal capsule; M1, primary motor cortex; MAS, modified Ashworth scale; MCS, motor cortex stimulation; NP, Neuropathic Pain; NR, not recorded; NRS, numerical rating scale; PSP, post-stroke pain; PVG, periventricular gray; SCS, spinal cord stimulation; VAS, visual analog scale for pain; VS, ventral striatum. Duration is defined by the time of the final follow-up for the study. Studies are coded by rigor scores: low (orange, 0–2), medium (green, 3–4), and high (blue, 5–6).

**Table 2:** Literature investigates non-invasive brain stimulation techniques for post-stroke pain.

Author, Year	Stim	Pain	Target	Parameters	Measures	Findings	AEs	Rigor Score
Wang et al., 2025 (27)	TMS *n* = 85	SP	M1 Erb’s Point	Sham controlled: Yes Number of sessions: 15 Frequency: 1, 10 Hz Pulse Number: NR Intensity: 120% resting MT Duration: 20 min	MAS FMA for Upper Extremity	Significant reduction in MAS Significant improvement in FMA Upper Extremity scores	No	6
Hosomi et al., 2013 (49)	TMS *n* = 21	CPSP	M1	Sham controlled: No Number of sessions: 1 Frequency: 5 Hz Pulse number: 500 Intensity: 120% resting MT Duration: 10 min (10s stimulation, 50s ISI)	Resting MT VAS	8 of 21 patients had ≥30% pain reduction Responders showed lower baseline intracortical facilitation compared to controls and nonresponders, which increased after rTMS	No	2
Chen et al., 2021 (112)	TMS *n* = 32	SP	Cerebellum	Sham controlled: Yes Number of sessions: 10 Frequency: 20 Hz-2 kHz Pulse width: 200–500 μs Intensity: 80% active MT Duration: 3.3 min	MAS Modified Tardieu Scale Shear Wave Velocity Motor-evoked Potential Barthel Index	Improvement in MAS, Modified Tardieu Scale, shear wave velocity, and Barthel Index in cerebellar iTBS compared to sham group	No	5
Kobayashi et al., 2015 (114)	TMS *n* = 18	CPSP	M1	Sham controlled: No Number of sessions: 12 Frequency: 5 Hz Pulse number: 500 Intensity: 90% active MT Duration: 10 min (10s stimulation, 50s ISI)	VAS Quick Inventory of Depressive Symptomatology	At week 12, 61.1% of participants were classified as responders: 5 showed >70% pain reduction, 6 showed 40–69% reduction, and 7 showed <40% reduction on the VAS	No	1
Hasan et al., 2014 (115)	TMS *n* = 14	CPSP	M1	Sham controlled: No Number of sessions: 5 Frequency: 10 Hz Pulse number: 2000 Intensity: 80–90% MT Duration: 20 min (10s stimulation, 50s ISI)	NRS Quantitative Sensory Test Brief Pain Inventory Hospital Anxiety and Depression Scale	Improved cold detection threshold Modest NRS pain reduction (from 7.0 to 6.3) Greater improvement in warm detection is associated with larger reductions in pain	No	1
Ohn et al., 2012 (116)	TMS *n* = 22	CPSP	M1	Sham controlled: No Number of sessions: 5 Frequency: 10 Hz Pulse number: 1000 Intensity: 90% resting MT Duration: 50 min (5 s stimulation, 55 s ISI)	fMRI VAS HDRS	14 responders showed significant reductions in VAS scores, lasting 2 weeks Lower baseline depression scores were associated with greater pain relief Higher ipsilesional superior thalamocortical tract integrity was correlated with greater VAS Decreased activity in secondary somatosensory cortex, insula, prefrontal cortex, and putamen	No	2
Ojala et al., 2022 (118)	TMS *n* = 17	CPSP	M1 S2	Sham controlled: Yes Number of sessions: 10 Frequency: 10 Hz Pulse number: 5050 Intensity: 90% MT Duration: 50 min (10s stimulation, 50s ISI)	NRS Cold Pressure Test Brief Pain Inventory Disabilities of the Arm, Shoulder, and Hand Pain Anxiety Symptom Scale Health-related QoL Beck Depression Inventory	rTMS to S2 produced ≥30% long-term pain reduction in 18% of participants All stimulations yielded similar short-term relief (~17–20%), likely placebo effect Only S2 stimulation showed significant long-term effects Cold pressor test revealed significantly reduced pain sensitivity	No	4
de Oliveira et al., 2014 (119)	TMS *n* = 21	CPSP	PMC dlPFC	Sham controlled: Yes Number of sessions: 10 Frequency: 10 Hz Pulse number: 1250 Intensity: 120% resting MT Duration: 12.5 min (5 s stimulation, 25 s ISI)	VAS Neuropathic Pain Questionnaire McGill Pain Questionnaire HARS HDRS 36-Item Short-form Health Survey	Active rTMS showed no significant advantage over sham stimulation across any of the assessed outcomes	No	5
Molero-Chamizo et al., 2021 (42)	tDCS *n* = 3	SP	M1	Sham controlled: Yes Number of sessions: 5 Intensity: 1.5 mA Duration: 20 min	VAS FMA	Marked or complete pain relief in 2 patients and partial improvement in 1 patient Spasticity improved significantly in 1 patient	No	3
Bae et al., 2014 (129)	tDCS *n* = 14	CPSP	M1	Sham controlled: Yes Number of sessions: 9 Intensity: 2 mA Duration: 20 min	VAS Pain from cold and heat Skin Temperature	Reduced VAS scores and skin temperature Increased cold pain/sensation thresholds Decreased heat pain/warmth thresholds	No	4
Halakoo et al., 2021 (131)	tDCS *n* = 32	SP	M1	Sham controlled: Yes Number of sessions: 10 Intensity: 2 mA Duration: 20 min	MAS EMG	Reduces wrist flexor spasticity Enhances muscle activity during movement	No	5
Ochi et al., 2013 (132)	tDCS n = 18	SP	M1	Sham controlled: Yes Number of sessions: 10 Intensity: 1 mA Duration: 10 min	MAS Motor Activity Log FMA	Improved FMA No significant change in Motor Activity Log Greater improvement in distal spasticity with tDCS + robot-assisted arm training	No	4
Del Felice et al., 2013 (133)	tDCS *n* = 10	SP	M1	Sham controlled: Yes Number of sessions: 5 Intensity: 1 mA Duration: 20 min	MAS Bhakta Finger Flexion Scale Postural Assessment Scale for Stroke Patients HDRS Passive Range of Motion Spinal excitability	Reduced spasticity, increased strength, and improved behavior Greater reduction in distal spasticity and longer-lasting proximal spasticity reduction with cathodal tDCS	No	4
Ehsani et al., 2022 (134)	tDCS *n* = 32	SP	M1	Sham controlled: Yes Number of sessions: 10 Intensity: 1 mA Duration: 20 min	Berg Balance Scale MAS (plantar flexors) EMG	Decrease in lateral gastrocnemius spasticity Increase in tibialis anterior activity Improved balance	No	5
Peng et al., 2026 (148)	taVNS *n* = 15	CPSP	Cymba Tragus	Sham controlled: Yes Number of sessions: 1 Frequency: 15 (cymba), 100 (tragus) Hz Intensity: 2x PT Duration: 30 min	Quantitative Sensory Test VAS	Thermal pain significantly improved following active taVNS VAS reduced in both active and sham taVNS groups	No	4

CPSP, central post-stroke pain; dlPFC, dorsolateral prefrontal cortex; EMG, Electromyography; FMA, Fugl-Meyer assessment; HARS, Hamilton anxiety rating scale; HDRS, Hamilton depression rating scale; M1, primary motor cortex; MAS, modified Ashworth scale; MT, motor threshold; NR, not recorded; NRS, numerical rating scale; PMC, premotor cortex; PSP, post-stroke pain; PT, perceptual threshold; SP, spasticity pain; S2, secondary somatosensory cortex; taVNS, transcutaneous auricular vagus nerve stimulation; tDCS, transcutaneous direct current stimulation; TMS, transcranial magnetic stimulation; VAS, visual analog scale for pain. Duration is defined by the length of an individual stimulation. Studies are coded by rigor scores: low (orange, 0–2), medium (green, 3–4), and high (blue, 5–6).

The primary objective of this review is to summarize and evaluate the current evidence for neuromodulation modalities for various forms of post-stroke pain specifically. The secondary objectives are to introduce the proposed underlying mechanism of post-stroke pain, as well as compare invasive and noninvasive modalities, highlight methodological limitations in the existing literature, and identify directions for future research. Although comprehensive, the narrative nature of this review was intended to provide broad and representative coverage of the field rather than to serve as a formal systematic review or meta-analysis, and it does discuss in some cases the impact of these techniques on other forms of pain for context (not included in the formal PubMed search). Overall, while the evidence for neuromodulation techniques in PSP remains very limited, it is an area of research that has shown promise, although more rigorous research is necessary.

## Post-stroke pain types and mechanisms of action

**Figure 1:**
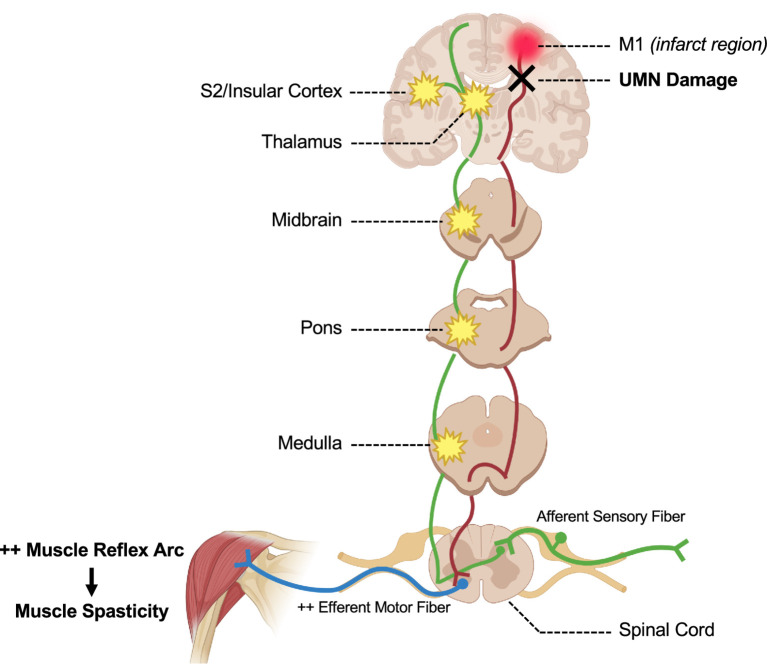
Overview of central post-stroke pain and spasticity pathways. The spinothalamic tract (green), responsible for conveying nociceptive and thermal signals to the brain, ascends from the dorsal horn of the spinal cord up through the brainstem to the thalamus and ultimately the cortex. Lesions along this tract (marked in yellow) can lead to CPSP, leaving the somatic sensory pathway intact. In comparison, the corticospinal tract (red) descends from the motor cortex through the internal capsule, with most fibers decussating primarily in the medulla to control voluntary motor function of the contralateral limbs. Following a stroke, upper motor neuron (UMN) damage can cause a dysregulation of inhibitory control of motor fibers, causing an overactive mechanical reflex arc, leading to muscle stiffness and spasticity.

PSP is a common complication in stroke survivors, and many patients experience daily pain (19). Additionally, stroke survivors with pain are at increased risk of functional dependence and psychiatric comorbidities (20, 21). Risk factors for post-stroke pain include ischemic stroke subtype and localized thalamic lesions (22). PSP is a heterogeneous set of syndromes like central post-stroke pain (CPSP) or muscle spasticity that can be present alone or in combination and may include nociceptive, mechanical, or neuropathic elements (22) (see Figure 1 for an overview of CPSP and spasticity).

Musculoskeletal pain often arises from biomechanical changes due to paralysis, spasticity, or contractures of the affected limb, and is therefore primarily mechanical and nociceptive in origin (23). Spasticity-induced musculoskeletal pain may develop from central lesions of the corticospinal tract, brainstem, or internal capsule, causing disinhibition of spinal reflex circuits (24). As a result of the decrease in the corticospinal activation, there is a dysregulation and ultimately resulting in excessive activation of descending excitatory motor pathways (14, 25, 26). While spasticity is due to central lesions or upper motor neuron damage directly from the stroke, contractures and other deformities can subsequently develop from immobility, limited range of motion, and compensatory movements or postures (24, 25, 27). For example, in hemiplegic shoulder pain, upper motor neuron damage leads to abnormal shoulder girdle alignment, altered biomechanics, ultimately resulting in spasticity and associated pain (28–31) (Figure 1, red pathway).

In contrast, neuropathic pain may arise from damage to the peripheral or central nervous system. In stroke, a common form of neuropathic pain arising as a direct consequence from the stroke lesion affecting the central somatosensory system, is central post-stroke pain (CPSP). Often, CPSP is caused from damage to the thalamus (most often the ventral posterior thalamus) or along the spinothalamic tract, including brainstem structures, leading to altered pain processing (14). However, it is also possible to have CPSP from cortical lesions, including the primary motor or insular cortices (32, 33). In CPSP, the pain and temperature signaling of the spinothalamic tract is disrupted while the dorsal column pathways are often spared (33). CPSP is thought to affect approximately 7–11% of stroke survivors, or around one out of five individuals who have PSP (7). The mechanism of CPSP has not been fully elucidated due to the complexity of central pain processing, but putative mechanisms are thought to include an imbalance between sensory pathways. For example, lesions in the spinothalamic tract can cause a disinhibition of pain signals to the anterior cingulate and insular cortices, causing the characteristic burning sensation and pain hypersensitivity (34, 35) (Figure 1, green pathway).

## Neuromodulation for post-stroke pain management

Common neuromodulation approaches for the management of PSP include invasive techniques such as DBS, MCS, SCS, and VNS, as well as non-invasive techniques such as TMS, tDCS, taVNS, and LIFU (13–16, 36–44). Overall, these neuromodulation techniques may help restore aberrant connectivity within the pain-processing networks, but the precise mechanisms appear to depend on the modality and target region. Even within a single brain stimulation modality (DBS), the authors found that multiple brain areas, ranging from the internal capsule to the thalamus, were effective at reducing pain in individuals with CPSP (45). Further, brain stimulation appeared to reduce hyperexcitability and restore connectivity patterns between these target substrates and the sensory cortex (45). In invasive modalities targeting thalamic nuclei, brain stimulation is thought to disrupt abnormal thalamocortical activity and reduce aberrant pain signals to the cortex. Neuroimaging studies corroborate that effective thalamic targeting in PSP patients modulates functional connectivity to cortical areas, including the insula, parietal lobe, and posterior cingulate cortex (26, 45). Electrophysiological studies show that DBS targeting the thalamus modulates thalamic neural oscillations in frequencies linked to pain perception, correlating with improved subjective pain (46, 47).

Another proposed mechanism includes restoring abnormal cortical excitation, especially in the primary motor cortex (M1), which is often hypoactive in CPSP (48). For example, increased M1 activity via neurostimulation has been shown to significantly correlate with the alleviation of pain (49). Moreover, it can modify connectivity, both structural and functional, in the thalamocortical and limbic circuits, specifically the amygdala, known to be critical in both the sensory and affective components of pain (26, 50–52). Finally, the somatosensory cortices have been implicated as areas of interest for alleviating PSP. In a nonhuman primate model, high-frequency rTMS enhances activity in the primary somatosensory cortex (S1) while suppressing the secondary somatosensory cortex (S2), suggesting a role for both S1 excitation and S2 inhibition in analgesia (51).

**Figure 2:**
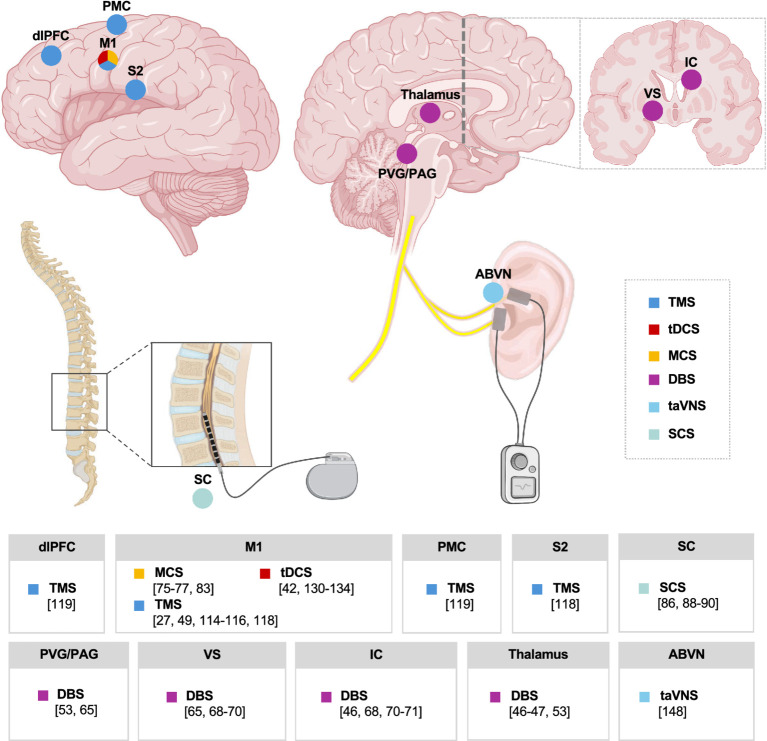
Invasive and non-invasive brain stimulation for post-stroke pain. A summary of the regions of interest for each study discussed in this review, including all forms of invasive brain stimulation, including spinal cord stimulation (SCS), vagus nerve stimulation (VNS), deep brain stimulation (DBS), and motor cortex stimulation (MCS), as well as non-invasive brain stimulation, including transcutaneous auricular vagus nerve stimulation (taVNS), low-intensity focused ultrasound (LIFU), transcranial magnetic stimulation (TMS), and transcranial direct current stimulation (tDCS), as it relates to post-stroke pain.

In the following sections, we review the current literature on invasive and non-invasive neuromodulation for PSP, discuss modality-specific mechanisms, and highlight future directions for the field (see Figure 2 for an overview of stimulation targets across all brain stimulation interventions and their corresponding references).

## Invasive neuromodulation techniques

There is ongoing research into the use of invasive neuromodulation techniques for multiple forms of PSP. While these techniques, including DBS, MCS, SCS, and VNS, can be highly beneficial, drawbacks such as substantial surgical risk and overall cost have made it such that these procedures are often limited to refractory cases (41, 53, 54). The indication, efficacy, and risks of each modality are discussed in the following sections, and a summary of the cited studies is presented in Table 1.

### Deep brain stimulation

Deep brain stimulation (DBS) is a procedure in which small burr holes are drilled directly into the skull and electrodes are placed into the brain parenchyma using stereotctic targeting (55). An implantable pulse generator is then placed in the chest wall, and a subcutaneous wire is tunneled from the skull to power the electrode lead (54, 56). DBS is currently FDA-approved for Parkinson’s disease, essential tremor, dystonia, epilepsy, and obsessive-compulsive disorder (OCD). Beyond these indications, numerous studies across multiple neuropathologies have also assessed its potential analgesic properties (13). For example, it has been shown to reduce subjective pain in patients with spinal cord injury (57–60), Parkinson’s disease (39, 61–63), and chronic neuropathic pain (41, 57, 60, 63, 64). The most common regions for targeting chronic pain include the thalamus and the periaqueductal/periventricular gray (PAG/PVG) (55, 65, 66). However, other neural targets continue to be explored, especially those critical for the affective component of pain. In fact, DBS targeting the anterior cingulate cortex (ACC) was shown to significantly improve affective components of pain in neuropathy, however overall pain scores did not improve (67).

Though less robust, there is a body of evidence investigating the impact of DBS on forms of PSP. For example, in a 12-year prospective study with patients who underwent DBS implantation to the thalamus and/or the periaqueductal gray (PAG), approximately 44% of patients experienced significantly improved quality of life, and the mean improvement in pain score (as measured by visual analogue scale, VAS) for the 14 patients that had follow up at the four year mark was 36% (53). A separate retrospective multicenter study observed benefits of DBS lasting up to the 12-month follow-up period. Their analysis of the most effective target region for pain alleviation indicated that the sensorimotor and medial intralaminar thalamic nuclei, as well as the posterior limb of the internal capsule (PLIC) had the highest rates of pain reduction in patients following implantation (45). A large systematic review article assessed PSP outcomes in 218 patients who received DBS in a variety of structures, including the thalamus, posterior limb of the internal capsule, or the PAG. Of note, patients who received stimulation of both the PAG and somatosensory thalamus tended to experience more pain relief than those in the thalamus alone (26). Additionally, the posterior insula and thalamus were associated with both pain-causing lesions as well as DBS targets used to alleviate pain in this population. Finally, the functional connectivity changes to areas implicated in affective pain processing distinguished lesions in responders versus non-responders to neuromodulation (26). However, the authors discussed that results should be taken with caution based on the large electrode insertional effect (57) and a lack of long-term follow-ups in the study.

The ventral striatum (VS) has also been a substrate of interest in this population. In a double-blind randomized study, 10 PSP patients had electrodes implanted into the VS/anterior limb of the internal capsule and received three months of active and sham stimulation in a crossover design (68). No significant improvement in Pain Disability Index was found, but other significant differences related to the affective component of pain, such as the Beck Depression Inventory, Affective Pain Rating Index, and McGill Pain Questionnaire, were observed between active and sham (68). In a unique experiment, PSP patients with DBS electrodes placed in the VS were given visual cues prior to receiving a painful or nonpainful stimulus to the affected or nonaffected limb, and researchers recorded event-related fields of pain anticipation using magnetoencephalography (MEG) (69). Active DBS stimulation significantly modulated early event potentials seen with MEG related to the anticipation of the painful stimulus compared to baseline or DBS-off conditions, suggesting improvements in the affective pain network and possible restoration of pain salience (69). Finally, in another study of the effect of VS stimulation, five PSP patients (in DBS ON and DBS OFF conditions) and five healthy controls received a heat stimulus, and neural activity was assessed via fMRI BOLD signals. Active DBS significantly reduced the activity of regions associated with the affective component of pain, including the thalamus, insula, orbitofrontal cortex (OFC), and hippocampus (70).

Additional studies exist examining the impact of DBS stimulation in other regions, but evidence is sparse. For example, a retrospective study of four patients with implanted DBS of the PLIC for CPSP. While one patient did not experience any change in pain, the other three patients were noted to have a significant reduction in acute pain one week after implantation. At nearly 6-year follow-up, the patients’ visual analogue scale (VAS) assessment of pain was still reduced from baseline with a mean long-term pain reduction of approximately 38% in all patients (71). In a case study of a single woman with refractory left hemibody pain following a stroke, electrodes were placed in the most common regions to target for pain, the PVG and the thalamus, but also the nucleus accumbens (NAc). Pain improvement was reported from individual stimulation of the PVG and NAc, but not the thalamus, and long-term pain reduction was maintained with long-term stimulation of both of these regions, implicating the NAc as a possible target for improving PSP (65). However, this conclusion has not been corroborated in a randomized controlled trial.

Based on these data, it is thought that DBS, depending on the target, may affect the sensory or affective pathways of pain. Targeting the thalamus and the PLIC may alter functional connectivity between the thalamus and sensory cortical areas, regulating any aberrant signaling following stroke (26, 45, 71). In contrast, studies that have targeted limbic structures such as the ventral striatum (69) have proposed that DBS can modulate the affective component of pain. Specifically, stimulation of these limbic regions may modulate activity in regions like the OFC, amygdala, and hippocampus, all known to be critical in pain perception and salience (65, 66, 68–70).

### Motor cortex stimulation

Motor cortex stimulation (MCS), a neuromodulation technique that dates to the 1990s for chronic pain, is an invasive modality in which electrodes are placed subdurally or epidurally to stimulate the motor cortex. In patients with multiple types of neuropathic pain, including trigeminal neuralgia and phantom limb pain, response rates have been seen as high as 60%, and long-term follow-up has indicated that clinically meaningful pain reduction can last years (40, 72–74). However, while one study found an approximately 41% probability of a significant pain reduction following MCS implantation, 39% of patients experienced a substantial post-operative analgesic effect following electrode insertion in the absence of stimulation, suggesting a strong placebo effect or expectation bias (73).

Although the data remain limited, small studies in patients with CPSP show similar results. A retrospective study of 21 individuals with CPSP who were treated with MCS assessed the impact of stroke location on stimulation efficacy. VAS of pain in individuals with thalamic stroke was reduced by 50% at follow-up (mean follow-up time = 65 months), and the neuropathic pain symptom inventory (NPSI) was significantly reduced compared to baseline (75). In those with extrathalamic strokes, however, the VAS was reduced by 25% and no significant change from baseline in NPSI was observed, suggesting MCS may have higher efficacy or durability in those who have suffered thalamic strokes (75). However, in two separate studies working to elucidate possible predictive variables for MCS in CPSP, neither stroke location (thalamic vs. extrathalamic) nor stroke type (ischemic vs. hemorrhagic) was found to be predictive of pain outcomes (74, 76, 77). Further, no difference was seen in epidural compared to subdural electrode placement for the reduction in CPSP (76, 78). Overall, a meta-analysis of a total of 32 studies with 191 patients who received MCS for treatment-refractory CPSP, the authors found a pooled responder rate of 64% and an overall mean pain score reduction of just over 50% (41). As there remains a paucity of randomized placebo-controlled trials assessing the impact of MCS on PSP, these benefits remain largely speculative.

MCS is currently thought to reduce the thalamic hyperactivity through modulation of thalamocortical pathways. In fact, the integrity of thalamocortical tracts between the afferent fibers from the thalamus and pyramidal cells was shown to be necessary in epidural MCS for its antinociceptive effects (79). Interestingly, it is thought that the analgesic effects of MCS are primarily from cathode induction of electrical changes in horizontal interneurons that run parallel to it, as opposed to the perpendicular pyramidal neurons (80). Overall, MCS is thought to activate descending pathways to restore inhibitory control over thalamic hyperexcitability to reduce pain, but evidence suggests it may also indirectly modulate the affective component of pain via the endogenous opioid system. Pain relief from MCS demonstrated a significant increase in endogenous opioid levels in the PAG and cingulate cortex (81). Further, preoperative levels of endogenous opioid binding to the PAG, insula, and thalamus were positively correlated with postoperative pain relief from MCS (82). One study demonstrated a decrease in the ratio of the affective and sensory scores in the McGill Pain Questionnaire following MCS, suggesting MCS may preferentially modulate the affective component of pain (83). And while MCS may be beneficial in treatment-refractory PSP, there are several limitations to this procedure. Beyond its invasive nature, its efficacy appears to be equivalent to less invasive methods for pain, and evidence for long-term efficacy remains limited, but in studies that did perform long-term follow-up, they often found that pain improvement diminished over time (84).

### Spinal cord stimulation

Spinal cord stimulation (SCS) involves the placement of a small stimulator in the epidural space over the spinal cord, often at the thoracic level T8-T10. However, the stimulator may be placed over the cervical spine for upper extremity pain. SCS is currently approved for chronic intractable pain of the trunk or limbs, including complex regional pain syndrome, radicular pain, peripheral neuropathy, and failed back surgery syndrome (85). Research on SCS for PSP to date remains limited. A case series of two patients suffering from stroke resulting in CPSP that had SCS implanted after failed medication management indicated that CPSP completely resolved following implantation (86). A separate case study also found SCS to be effective in the alleviation of hemibody CPSP (87). And in a sixty-year-old female with a right-sided thalamic stroke suffering from CPSP of her left hemibody that was refractory to multiple medication trials, percutaneous SCS was placed at T5-T6. Based on a numeric pain rating scale, the patient experienced approximately a 50% reduction in pain 6 days post-procedure and maintained until the one-month follow-up (88). In a retrospective multicenter study of 166 patients with CPSP who underwent SCS placement, (89) researchers assessed pain intensity and patient global impression of changes (PGIC) for the patient’s perceived improvement. The mean pain score decreased by an average of 42, and 56% reported much or very much improved pain based on the PGIC. Additionally, 41% of patients maintained their improvements at a median follow-up time of 24 months (89). To understand the predictive factors of the success of SCS for CPSP, Tanei and colleagues (90), via a retrospective review, assessed 18 patients who underwent SCS visual analog scale (VAS) of pain preoperatively, and at 1-, 6-, and 12-month post-SCS placement. Significant associations were found between pain improvement and both age and stroke location, suggesting SCS is more effective in reducing CPSP in younger patients and those with non-thalamic strokes (90).

The proposed mechanism of action for SCS in the treatment of PSP involves the modulation of segmental and supraspinal pathways. At the level of the spinal cord, SCS has been shown to modulate pain via the gate control theory, which states that stimulation activates large-diameter dorsal column Aβ-fibers that inhibit nociceptive signaling from smaller fibers in the dorsal horn via the release of GABA and endocannabinoids (91). Supraspinally, SCS modulates brain regions involved in pain perception and affect, including the thalamus, anterior cingulate, and prefrontal cortices (43). Finally, emerging evidence in neuropathic models suggests that SCS may attenuate microglial activation within the spinal cord, thereby reducing neuroinflammation associated with neuropathic pain (92).

There are several issues that must be considered with SCS as it relates to post-stroke pain. Overall, studies have noted a high device failure rate, leading to explantations of the device. As such, if SCS is to be pursued, a trial period prior to permanent implantation is critical (89). And while there appears to be improvement with SCS for CPSP, the response rate appears higher for complications from peripheral neuropathic pain syndromes, such as failed back surgery syndrome or complex regional pain syndrome (36). Finally, there appears to be more variability in pain improvement in SCS than in other modalities (15). For example, a meta-analysis of SCS for neuropathic pain showed some improvement, but with considerable heterogeneity across studies and efficacy ranging from 38 to 89% depending on condition and patient population (93, 94).

### Vagus nerve stimulation

Invasive vagus nerve stimulation (VNS) is a neurosurgical procedure where stimulating electrodes are wrapped around the vagus nerve for stimulation, and is currently FDA-approved for drug-resistant epilepsy, treatment-resistant depression, migraine or cluster headaches, and stroke motor rehabilitation (95). In one study, researchers investigated several forms of experimental pain in patients with treatment-resistant epilepsy before and after VNS implantation, compared to age and gender-matched controls, and found that VNS significantly reduced pain to stimuli specifically modulated via central processes (96). While VNS stimulation may alter pain sensation peripherally, the antinociceptive effects appear more pronounced when pain is amplified by central mechanisms (97).

VNS has also been explored for use in multiple disorders of chronic pain, such as fibromyalgia, pelvic pain syndrome, and headaches (98–101). However, the magnitude and consistency of these antinociceptive findings vary (102, 103). VNS has been FDA-approved for use in stroke patients for upper extremity function in conjunction with rehab exercise (37, 104, 105). A meta-analysis of eight studies using VNS (4 implanted, 4 transcutaneous) found that stimulation increased upper limb function, as assessed by the Fugl-Meyer scale, by 7 points compared to approximately 2.5 in the control intervention of rehab alone (106). These findings should be interpreted cautiously, as the available pain data for VNS come from non-PSP populations. To date, its analgesic effects have not been directly established in individuals with post-stroke pain.

Like other stimulation modalities, VNS is thought to inhibit spinal nociceptive pathways. However, it has also been shown to have anti-inflammatory effects that may improve pain. Preclinical studies examining VNS have demonstrated a release of acetylcholine, which binds *α*7-nicotinic acetylcholine receptors (α7nAChR) on immune cells (107). As a consequence, there is both a reduction of pro-inflammatory cytokines (particularly TNF-α, IL-1β, and IL-6), as well as increased levels of the anti-inflammatory cytokine IL-10 (107–109). In addition, afferent VNS appears to reduce lipopolysaccharide (LPS)-induced inflammation independently of immune-cell-derived acetylcholine release that appears to drive efferent signaling (110). To date, no research has specifically examined the impact of taVNS on PSP.

Invasive stimulation for post-stroke pain remains promising but incompletely understood. Available data suggest that DBS, MCS, and SCS can reduce pain in selected patients, potentially through effects on sensory-discriminative and affective pain networks, yet these findings are drawn largely from case reports, retrospective cohorts, and meta-analyses of low-quality studies rather than robust randomized trials. As a result, these approaches are generally reserved for treatment-refractory cases, and firm conclusions about comparative efficacy, durability, and optimal patient selection cannot yet be made.

## Non-invasive neuromodulation techniques

An increasing number of studies have worked to understand the impact of multiple non-invasive brain stimulation (NIBS) techniques, including TMS, tDCS, taVNS, and LIFU, on neuropsychiatric conditions as effective alternatives to their invasive counterparts. The following sections will discuss NIBS modalities as they relate to PSP, and an overview of the studies cited can be found in Table 2.

### Transcranial magnetic stimulation

Transcranial magnetic stimulation (TMS) is a non-invasive technique that uses magnetic pulses to generate an electric field superficially at the level of the cortex to alter neural excitability. Currently FDA-approved for depression, obsessive compulsive disorder, and smoking cessation, it has also been used experimentally for some chronic pain conditions, including fibromyalgia, complex regional pain syndrome, and other neuropathies (18, 111). Specifically, repetitive transcranial magnetic stimulation (rTMS) was assessed for its impact in managing upper extremity (UE) spasticity and motor function following stroke. In a large, randomized placebo-controlled study, 110 patients with UE spasticity following stroke were randomly assigned to daily 20-min sessions of either active or sham rTMS to the M1 for a total of 15 sessions over the course of six weeks, in conjunction with traditional motor rehabilitation. Following stimulation, the active group had a significant reduction in the Modified Ashworth Scale (MAS), an assessment of muscle tone (27). And while functional scores significantly improved in both the active and sham groups following stimulation, the improvement in the active group was significantly higher compared to the sham group. Overall, these results suggest rTMS can improve spasticity and motor function (27). In another study of 32 patients with UE spasticity following stroke, participants received either intermittent theta burst (iTBS) or sham stimulation for 2 weeks prior to conventional therapy. Patients treated with active iTBS had improved motor-evoked potentials (MEP) in the UE, as well as reduced muscle spasticity as measured by the MAS, suggesting it is a possible adjuvant of conventional therapy to improve spasticity following stroke (25, 112). Finally, a review assessing 14 total studies exploring the impact of TMS on post-stroke spasticity found that stimulation to the motor/premotor cortices resulted in significantly improved MAS scales across all studies (113).

As it relates to CPSP, current TMS research has primarily focused on the primary motor cortex (M1) for pain management, similar to MCS. In one small pilot study, 18 stroke patients received rTMS at M1 on the affected side once a week for 12 weeks, with six of the patients continuing for one year. At week 12, 11 out of 18 patients had at least a 40% reduction in pain, and pain relief was maintained for those patients who continued for a year (114). Hasan and colleagues utilized MRI mapping to target 14 patients’ pain hotspots within the M1 that correlated to a painful region in an open label trial. Targeted rTMS using 2000stimuli/10 Hz per session for 5 total sessions was used, and quantitative sensory threshold (QST) testing was performed to assess changes in heat/cold pain detection and thresholds (115). A significant improvement in thermal detection was observed, which also correlated with improved subjective pain scores (115). Similarly, a study using MR-guided TMS over the hotspot on the interosseous muscle for 1,000 pulses at 10 Hz for 5 consecutive days assessed subjective pain (VAS) as diffusion tensor imaging and fMRI to assess changes in the pain network (116). Pain significantly decreased in 14 of 22 patients stimulated, which was maintained for 2 weeks following stimulation. Interestingly, a decrease in activity in the secondary somatosensory cortex (S2), insula, prefrontal cortex, and putamen was found following stimulation in responders compared to non-responders, suggesting pain attenuation from TMS may be due to an alteration in the pain network more broadly (116).

However, the specific impact of TMS within the pain pathway remains under debate. One study using TMS for chronic deafferentation pain, nerve damage that can result from stroke, spinal cord injury, or other neurological conditions, performed rTMS at the M1, as well as the somatosensory cortex (S1), premotor area, and supplementary motor area (SMA) (117). Each target was stimulated with 10 trains of 10s pulses of 5 Hz, therefore receiving a total of 500 stimulations with sham stimulations randomly interspersed. Results showed that pain, as measured by VAS and the McGill Pain Questionnaire, was significantly reduced in 10/20 patients when M1 was targeted, lasting up to 3 h post-stimulation. However, no other region had any impact on pain, even though these regions are adjacent and strongly connected to M1 (117). It is important to note that this study was performed in a heterogeneous population and therefore, conclusions for PSP specifically remain opaque. Conversely, a sham-controlled three-arm crossover trial directly compared rTMS targeting M1 versus S2 in individuals with PSP. They found that all groups (sham, M1, and S2) showed an acute attenuation of pain, but only S2 maintained significant long-term pain relief at one month (118). In a unique sham-controlled study investigating the role of the dorsolateral prefrontal cortex (dlPFC), de Oliviera et al. (119) stimulated 21 patients with CPSP with active or sham TMS at the dlPFC with one session daily for ten sessions. Changes in pain, as assessed by a VAS, were unchanged immediately following, as well as one-, two-, and four-week post-stimulation (119).

Like invasive MCS, TMS targeting the M1 is thought to increase cortical excitability, ultimately normalizing dysregulated thalamic and somatosensory pathways damaged following stroke (89). There is also some evidence that TMS can normalize functional connectivity in regions critical for pain and emotion, namely the insula and cingulate cortex (52). The strength and durability of the effects of TMS for pain vary across studies depending on target location, dose, and duration (120–122). Multiple meta-analyses examining the impact of TMS on CPSP have shown significant pain reduction both immediately and 3 weeks post-stimulation (120, 123), with some studies suggesting the effect can last up to three months (124). However, it also appears the placebo effect may play a large role in these findings, particularly acutely post-stimulation. Notably, rTMS applied to the M1, S2, as well as sham stimulation, all produced significant improvements in subjective pain ratings immediately following stimulation (118). Further, a meta-analysis of nine studies found that sham TMS often has comparable acute effects on PSP as active stimulation, bringing the quality of current evidence of efficacy into question for the authors (120). Some studies suggest that high-frequency rTMS, shown to more likely increase cortical excitability than low frequency, can effectively alleviate PSP, and multiple sessions with longer durations have superior outcomes (123, 125). Although some early data are promising, further study is needed to define the optimal stimulation target, frequency, pulse dose, and treatment duration, as these parameters vary substantially across studies and appear to influence both efficacy and durability. Larger sham-controlled trials are also needed to determine maintenance schedules and distinguish true treatment effects from placebo response.

### Transcranial direct current stimulation (tDCS)

Transcranial direct current stimulation (tDCS) utilizes a small electrical current that flows between a cathodal and anodal electrode placed on the scalp to modulate the likelihood of cortical neuronal firing. While not FDA-approved for any neuropsychiatric condition at this time, it has been studied in conditions ranging from Parkinson’s disease to various subtypes of pain (126). Current evidence suggests that anodal tDCS increases cortical excitability, restoring disrupted interhemispheric balance and enhancing the activity of descending inhibitory pain pathways (127). This modulation can reduce maladaptive network reorganization and interhemispheric inhibition that contribute to CPSP (42). Additionally, tDCS may decrease intracortical inhibition, promote cortico-cortical excitability, and facilitate long-term potentiation, which can improve pain (128, 129). However, the overall clinical impact varies. A seminal study by Fregni et al. (130) using tDCS in spinal cord injury patients with chronic neuropathic pain found that 5 days of anodal 2 mA stimulation placed over M1 with cathodal placement over the contralateral supraorbital area significantly reduced pain in active stimulation compared to sham by the third day of stimulation and continued until the fifth day. However, pain reduction was not maintained at the 3-week follow-up (130). A meta-analysis of 27 studies evaluating tDCS for chronic pain corroborates the heterogeneity of impact of stimulation, with only a minimal clinical difference between active and sham groups, and no statistical difference in overall quality of life (18).

As it relates to specific forms of PSP, multiple randomized controlled trials have shown the benefit of tDCS for spasticity-related pain. In one study, 32 stroke patients were assigned to one of three groups: active anodal tDCS, sham, or a control functional electrical stimulation (FES). Stimulation was then delivered over the affected M1 for 20-min sessions for 10 total sessions. The Modified Ashworth Scale (MAS), a clinical measurement of spasticity, and electromyography (EMG) of the flexor and extensor carpi radialis muscles were analyzed before, immediately post, and at 1-month follow-up (131). Researchers saw a significant reduction in both MAS score and EMG activity immediately and at the one-month follow-up in the active compared to the sham and FES control groups (131). In an effort to elucidate the importance of electrode placement, a small crossover study with 18 patients recovering from upper extremity (UE) paresis secondary to stroke received two different treatments for five days each: anodal tDCS on the affected primary motor cortex (M1) with robot-assisted arm training, and cathodal tDCS on the unaffected M1 with the same training (the other electrode being placed over the contralateral supraorbital area) (132). Both interventions demonstrated equal improvements in multiple clinical movement scales, as well as MAS. One notable difference found was that cathodal tDCS significantly improved distal spasticity for right hemisphere but not left hemisphere lesions (132). Another crossover pilot study examined the impact of dual tDCS on UE spasticity. This experiment used anodal tDCS over the affected M1 with cathodal stimulation placed on the unaffected M1, with all patients receiving 20-min sessions for 5 consecutive days of sham, followed by stimulation with either cathodal or dual tDCS. Both tDCS protocols were shown to decrease spasticity, but cathodal stimulation was slightly superior in reducing spasticity compared to dual tDCS treatment in initial spasticity improvement and durability of response (133). Finally, in a randomized placebo-controlled trial examining lower extremity (LE) spasticity following stroke, patients received active or sham anodal tDCS to the affected M1 with concurrent physical therapy or physical therapy alone, with assessments taken immediately following and one-month post-stimulation. A significant reduction in MAS and EMG activity in the lateral gastrocnemius muscle was found both immediately and at the one-month follow-up time point (134). Overall, preliminary evidence suggests tDCS can significantly improve spasticity and associated pain, however the impact of anodal compared to cathodal stimulation remains a subject of debate (113, 132).

The evidence for tDCS-induced analgesia for CPSP is less conclusive. In a small, sham-controlled study of participants with CPSP (n = 14), tDCS was administered for 20 min at 2 mA current intensity with anodal stimulators on the primary motor cortex (M1) three days per week for three weeks demonstrated that subjective pain scores significantly decreased (129). In addition, an increase in the threshold temperature for cold sensation was observed, while also showing an increase in cold pain threshold in the active group, while no such change was seen in the sham. The authors conclude that anodal tDCS may have both an analgesic effect and improve sensory identification or threshold (129). A series of case studies exploring the effect of anodal tDCS targeting the contralateral M1 for upper extremity pain following stroke showed pain reduction in 2/3 patients who received stimulation, while spasticity was improved in 1 out of 3 patients, suggesting an analgesic effect but also highlighting substantial inter-individual variability (42). A separate case report examined the impact of anodal tDCS targeting another region, the dlPFC, in a post-stroke patient with two protocols. Specifically, the first involved 20 min of stimulation, 5 days per week. After a three-week washout phase, the second consisted of 7 daily sessions of 13-min stimulations each, separated by 20-min intersession intervals, over one week (135). The patient had significantly attenuated pain and depression scores immediately post-stimulation in the first protocol, but both rebounded to baseline levels at the three-week follow-up date (135). In contrast, following the second protocol, the patient had their pain scores decrease to zero and were maintained through until the six-month follow-up (135). The current evidence for the use of tDCS in PSP appears to show an analgesic effect, but this may be dependent on the underlying cause of the pain and stimulation type. Pilot studies must be replicated in larger, randomized controlled trials. Further, more research is needed to understand optimal anodal and cathodal electrode placement, as well as to compare the efficacy based on the location of the stroke lesion, to work to explain the strong variability observed in outcomes.

### Transcutaneous auricular vagus nerve stimulation

The non-invasive counterpart to VNS, transcutaneous auricular VNS (taVNS), targets the auricular branch of the vagus nerve via electrodes placed on the cymba conchae and tragus of the outer ear (136–138). taVNS has been shown to have positive effects experimentally in many neuropsychiatric conditions, such as depression, epilepsy, and post-stroke motor recovery (16, 17, 139–142). It also significantly increases the conditioned pain response, a proxy of descending pain pathways, in healthy subjects after a single stimulation session (143). Additionally, taVNS has demonstrated some analgesic effects in conditions like migraine (16, 98, 136). In addition to brainstem modulation and anti-inflammatory changes seen with VNS, mouse models of neuropathic pain have suggested the serotonergic system is critical in the analgesic effect of taVNS. The preclinical models have demonstrated a taVNS-induced increase in activity with the dorsal raphe nucleus, a critical node for serotonin release (144). Subsequently, the use of a serotonin synthesis inhibitor abolished the analgesic effects of taVNS (144).

taVNS has been shown to be safe, feasible, and well-tolerated overall in stroke patients (145), with little to no adverse events noted (146). Further, a randomized control trial evaluated the impact of taVNS on infarct growth in patients with acute stroke or intracerebral hemorrhage and showed that the relative growth of ischemic lesions on diffusion weighted imaging (DWI) 24 h following insult was 63% greater than baseline compared to 184% greater in the sham group (*p* = 0.109) (147). The authors suggest taVNS may have a neuroprotective effect when used in acute stroke (147). However, there is currently very limited research regarding the impacts of taVNS in post-stroke pain. In a small sham-controlled pilot study, our lab assessed the impact of a single 30-min stimulation session on PSP (148). Patients were stimulated using 15 Hz at the cymba concha and 100 Hz anterior to the tragus, with a pulse duration of 250 μs, while sham stimulation used identical parameters to those applied at the cymba concha but was delivered to the earlobe, an area of the ear that is less innervated by the vagus nerve. Following stimulation, patients in the active stimulation group had a statistically significant increase in pain threshold as assessed by quantitative sensory testing (QST) compared to the sham group, suggesting an antinociceptive effect of taVNS. This proof-of-concept pilot did not assess the durability of taVNS on pain, focusing more on the immediate antinociceptive effects. Further, it is likely a single session of taVNS is inadequate for significant lasting changes to pain. Although interest in taVNS has increased due to its ease and non-invasive nature, there remains a need for research into optimal stimulation parameters and large-scale trials to best assess the impact of taVNS on post-stroke pain (149).

### Low intensity focused ultrasound

Low-intensity focused ultrasound (LIFU) is a non-invasive stimulation technique that has garnered substantial interest in recent years due to its ability to perform both deep and focal stimulation at a circuit-specific level, precision that was previously restricted only to invasive techniques. Unlike its high-intensity counterpart, LIFU does not cause permanent ablation via thermal effects. It is currently used in research for emerging clinical applications for drug delivery and numerous neuropsychiatric conditions. There has been some initial investigation into the impact of LIFU on pain (150). For example, Song and colleagues, in an animal model of neuropathic pain using a chronic constriction model, found LIFU to the L5 level of the spinal cord significantly reduced allodynia as measured by the von Frey test (151). Further, LIFU was found to significantly attenuate microglial activation in the M1 region, suggesting LIFU improves neuropathic pain in part via an anti-inflammatory mechanism (151).

While clinical studies are limited, the preliminary results show promise. In one study of healthy volunteers, Strohman et al. (152) applied a transient heat-pain stimuli during either active or sham LIFU to the dorsal anterior cingulate cortex (dACC), and multiple subjective and objective outcomes were measured. Active LIFU significantly reduced subjective pain ratings compared to sham, while also altering contact heat evoked potential (CHEP) and heart rate variability (HRV). Their results indicate that LIFU targeting the dACC reduces pain and alters autonomic responses to acute heat stimuli (152). In another preliminary study, Legon and colleagues (153) used LIFU to compare the impact of modulation of the anterior (AI) and posterior (PI) insula on pain rating, HRV, and EEG activity to a painful heat stimulus in healthy subjects. While LIFU to both the AI and PI reduced pain ratings compared to sham, stimulation of the PI appeared to impact EEG amplitudes earlier compared to the AI. Further, only LIFU to the AI caused an increase in HRV (153). A separate paper by Badran et al. (154) assessed the impact of MRI-guided active or sham LIFU to the right anterior thalamus in 19 healthy individuals for two 10-min sessions (fundamental frequency: 650 kHz, Pulse repetition frequency: 10 Hz, Pulse Width: 5 ms, Duty Cycle: 5%). Quantitative sensory testing (QST) was used to assess changes in changes to sensation, pain, and tolerance of thermal stimuli. Thermal pain sensitivity was significantly attenuated following stimulation, suggesting an antinociceptive effect of the thalamus-targeted LIFU in healthy individuals (154).

Unfortunately, current research using LIFU in chronic pain populations is extremely limited. In one pilot, 31 individuals with chronic back pain underwent either active subthermal transcranial ultrasound (8 MHz) or placebo stimulation targeting the contralateral frontotemporal area in a double-blind crossover trial, with pain assessed 10 min prior to and 10 and 40 min following a single stimulation (155). Subjective mood was significantly improved at both 10- and 40-min post-stimulation compared with placebo. However, subjective pain reports, while they showed a trend, did not reach significance. (*p* = 0.07) at 40 min (155). Another randomized crossover trial of 20 patients with unspecified chronic pain received a 40-min session of active and sham LIFU in random order directed at the ACC, and a clinically meaningful decrease in pain (as assessed by the Brief Pain Inventory, BPI) immediately (60% decrease), and one (43% decrease) and seven (33% decrease) days following the active stimulation compared to sham (156). Finally, a recent pilot examined the impact of seven days of LIFU in diabetic neuropathy patients on the peripheral nerves (157). Compared to baseline, both active and sham groups showed significant improvements in pain scores, but the active group also demonstrated significantly lower scores compared to the sham group. Further, only the active LIFU group demonstrated significantly improved neuropathy symptom scores following the seven days (157). Overall, LIFU has been found to be safe and tolerated in several pain populations, but no research to date has examined its impact on PSP directly (150).

Despite its growing interest, the exact mechanism of action of LIFU neuromodulation continues to be debated. Compared to high-intensity focused ultrasound (HIFU), in which the sonication is ablative due to the significant temperature change at the region of interest, thermal effects do not appear to be the cause of changes in neuronal excitability (38, 158). Mechanisms like acoustic cavitation, where local pressures drop below the vaporization point of the lipophilic component of the cell membrane and cause bubbles that change membrane capacitance or permeability, as well as alterations of the excitement of specific mechanosensitive ion channels, have been proposed (38, 159, 160). However, the mechanism by which LIFU modulates PSP specifically remains unexplored.

Non-invasive neuromodulation for post-stroke pain shows early promise, particularly for TMS and tDCS, which have demonstrated analgesic effects in some studies and may also improve spasticity-related pain. taVNS is highly feasible and well tolerated and has shown proof-of-concept antinociceptive effects, while LIFU remains especially preliminary, with no direct studies yet in post-stroke pain. Overall, however, the literature is still limited by small sample sizes, pilot designs, heterogeneous stimulation parameters, and minimal follow-up, making it difficult to draw firm conclusions about durability or comparative efficacy. More rigorous, larger-scale studies are needed before these modalities can be considered established treatments.

## Conclusion and future directions

Overall, both invasive and non-invasive neuromodulation techniques demonstrate promising effects on reducing post-stroke pain, although the degree and consistency of improvement vary across studies (18, 122). Some evidence suggests that invasive stimulation techniques, particularly DBS, may produce more robust and sustained analgesic effects in patients with refractory central post-stroke pain (CPSP) (33, 49, 79, 80). Conversely, research has also shown that non-invasive brain stimulation (NIBS) techniques may reduce the impact of some types of post-stroke pain at similar levels as invasive modalities, without the significant surgical risks (10, 18, 22, 106, 122, 124). As such, utilization of safer, more readily available forms of NIBS makes them attractive first-line options for pain modulation in many cases. Importantly, the substantial variability in stimulation parameters, target regions, patient selection criteria, and outcome measures makes direct comparisons difficult between different forms of neuromodulation. Large-scale, well-controlled clinical trials directly comparing invasive and non-invasive techniques are needed to aid in clinical decision-making.

The use of brain stimulation techniques, particularly NIBS, is a burgeoning field with many lingering questions. Future research should focus on attempting to optimize stimulation protocols in PSP patients, focusing on outcomes depending on the type of pain being evaluated. Additionally, while some studies have shown spasticity and pain improve with a combination of neuromodulation, particularly TMS (25, 27, 113), and motor rehabilitation, other research demonstrates TMS alone is able to improve pain outcomes (10, 52, 70, 111, 116, 118). There is limited evidence comparing brain stimulation alone versus a combined treatment with rehabilitation for pain outcomes, making large-scale studies comparing these approaches essential for optimizing clinical outcomes. Finally, mechanistic studies using advanced neuroimaging and neurophysiological assessments will be critical for understanding how neuromodulation alters pain-processing networks post-stroke, guiding personalized and precision-based interventions in this challenging clinical domain (14, 16, 26, 55).
